# Ambient Temperatures in the Burn Operating Room

**DOI:** 10.1177/22925503231169759

**Published:** 2023-04-17

**Authors:** Alan Rogers, Syena Moltaji, David Wallace, Robert Cartotto

**Affiliations:** 1 Division of Plastic, Reconstructive and Aesthetic Surgery, Department of Surgery, University of Toronto, Toronto, Ontario, Canada; 2 Ross Tilley Burn Centre, 71545 Sunnybrook Health Sciences Centre, Toronto, Ontario, Canada

To the Editor,

Patients with major burn injuries are particularly susceptible to hypothermia (core temperatures below 36 °C) due to the disruption of their epidermal barrier.^1,2^ Cool ambient temperatures increase the metabolic rate and resting energy expenditure, diverting resources away from wound healing. Furthermore, perioperative hypothermia, specifically, has been shown to predispose to both infectious and non-infectious complications, including myocardial infarction, thromboembolic events, surgical site infections, and bleeding.^1-4^ Surveys suggest that one of the most important strategies to reduce perioperative hypothermia is to increase ambient temperatures in the operating room.^3,5^ As part of a quality improvement (QI) initiative, we sought to document these temperatures at an American Burn Association-verified burn centre, and establish the relationship, if any, between operating room ambient temperatures, operative duration, and our patients’ core temperatures at the conclusion of surgery.

We measured operating room temperatures for a one year period between March 1, 2020 and February 28, 2021, using a wall-mounted, smart sensor (Sensor Push^TM^, Garrison, New York, USA). This calibrated device collected temperatures at 1-min intervals and was synchronized via Bluetooth to an encrypted mobile smart phone using the Sensor Push^TM^ application, and then transferred the data to Microsoft Excel^TM^ for analysis. All acute burn surgeries over 2 h in duration were included in this QI study. Core patient temperatures are routinely measured at the end of surgery using a nasopharyngeal thermometer, and are recorded in a performance improvement database.

During the period of study, 119 patients underwent 261 acute burn surgeries longer than 2 h by 4 burn surgeons (mean 2.19 procedures per patient, range 1-15). The mean age of the patients was 49.82 years (range 18-82), the mean total body surface area (TBSA) burn was 15.82 (range 1%-62%), and most were male (n  =  86, 72.3%). The mean operative duration was 211 min (range 120-468). Eight patients died (6.7%, mean age 65 years).

There were 33 hypothermic patients at the end of 43 cases (16.48%). The number of operating room ambient temperature readings recorded was 34,944, with a mean temperature of 24.6 °C (range 18.73-29.62 °C). The mean room temperature during hypothermic cases was 23.9 °C, compared to 24.7 °C during normothermic cases (*P*  =  .009) (Table 1). The mean starting ambient temperature was also significantly lower: 22.8 °C versus 23.7 °C (*P*  =  .007). Surgeries starting earlier in the day were more frequently associated with hypothermia (Table 2). The rates of hypothermia differed between the 4 surgeons; these were associated more closely with the room temperature rather than their mean operative duration (Table 3).

**Table 1.** Relationship Between Patient Temperature, at the End of Surgery, With the Room Temperature, TBSA, Age, and Duration of Surgery.

	Cases (n)	Mean room temperature (°C)	Start room temperature (°C)	End room temperature (°C)	Total body surface area (%)	Age (years)	Surgery duration (min)
End patient temperature (<36 °C)	43	23.9	22.8	24.5	32.3	48.2	225.6
End patient temperature (≥36 °C)	218	24.7	23.7	25.1	24.8	49.4	208.3
Total	261	24.6	23.6	25.0	26.0	49.2	211.1
*P*-value		.009	.007	.058	.015	.36	.063

**Table 2.** Relationship Between the Procedure Start Time and the Incidence of Hypothermia.

	Normothermia (n)	Hypothermia (n) [%]	Cases (n)
Start before 10 am	98	25 (20.3)	123
Start after 10 am	120	18 (7.2)	138
Total	218	43 (16.48%)	261

**Table 3.** Relationship Between the Operating Surgeon, Mean Operative Duration, Incidence of Hypothermia and the Room Temperature.

Surgeon	Total cases (n)	Mean duration (min)	Hypothermic cases (n)	Mean room temperature (°C)	Start room temperature (°C)	End room temperature (°C)
#1	69	224.48	13 (18.84%)	23.85	23.38	24.12
#2	51	183.59	16 (31.37%)	24.07	23.15	24.59
#3	70	192.94	6 (8.54%)	24.98	23.81	25.43
#4	71	235.87	8 (11.27%)	25.23	23.97	25.77
All	261	211.1	43 (16.48%)	24.6	23.6	25.0

Patients with major burn injuries are more likely to develop intraoperative hypothermia with larger TBSA burns, longer surgeries, and when operated earlier in the day. Moderate differences in hypothermia rates between surgeons present opportunities for QI interventions, especially forcing functions like automation of operating room temperatures, especially earlier in the day. Patients with major burns benefit from a protocol that emphasizes perioperative warming with external warming devices, discussion of temperature at the surgical checklist, increasing ambient room temperature, and covering exposed, non-operated parts.^3-7^
